# Early Identification of HIV: Empirical Support for Jail-Based Screening

**DOI:** 10.1371/journal.pone.0037603

**Published:** 2012-05-25

**Authors:** Alex de Voux, Anne C. Spaulding, Curt Beckwith, Ann Avery, Chyvette Williams, Lauren C. Messina, Sarah Ball, Frederick L. Altice

**Affiliations:** 1 Rollins School of Public Health, Emory University, Atlanta, Georgia, United States of America; 2 Alpert Medical School of Brown University, Providence, Rhode Island, United States of America; 3 Case Western Reserve University School of Medicine, Cleveland, Ohio, United States of America; 4 School of Public Health, University of Illinois, Chicago, Illinois, United States of America; 5 Abt Associates Inc., Cambridge, Massachusetts, United States of America; 6 Section of Infectious Diseases, Department of Medicine, Yale University School of Medicine, New Haven, Connecticut, United States of America; 7 Division of Epidemiology of Microbial Diseases, Yale University School of Public Health, New Haven, Connecticut, United States of America; McGill University AIDS Centre, Canada

## Abstract

**Background:**

Although routine HIV testing is recommended for jails, little empirical data exist describing newly diagnosed individuals in this setting.

**Methods:**

Client-level data (CLD) are available on a subset of individuals served in EnhanceLink, for the nine of the 10 sites who enrolled newly diagnosed persons in the client level evaluation. In addition to information about time of diagnosis, we analyzed data on initial CD4 count, use of antiretroviral therapy (ART), and linkage to care post discharge. Baseline data from newly diagnosed persons were compared to data from persons whose diagnoses predated jail admission.

**Results:**

CLD were available for 58 newly diagnosed and 708 previously diagnosed individuals enrolled between 9/08 and 3/11. Those newly diagnosed had a significantly younger median age (34 years) when compared to those previously diagnosed (41 years). In the 30 days prior to incarceration, 11% of those newly diagnosed reported injection drug use and 29% reported unprotected anal intercourse. Median CD4 count at diagnosis was 432 cells/mL (range: 22–1,453 cells/mL). A minority (21%, N = 12) of new diagnoses started antiretroviral treatment (ART) before release; 74% have evidence of linkage to community services.

**Conclusion:**

Preliminary results from a cross-sectional analysis of this cohort suggest testing in jails finds individuals early on in disease progression. Most HIV^+^ detainees did not start ART in jail; therefore screening may not increase pharmacy costs for jails. Detainees newly diagnosed with HIV in jails can be effectively linked to community resources. Jail-based HIV testing should be a cornerstone of “test and treat” strategies.

## Introduction

Despite extensive prevention efforts and recommendation for routine HIV testing, there has been no substantial reduction in the 50,000 new HIV infections annually in the United States [1], [2]. Of the 1.1 million HIV-infected individuals in the United States, 20% are unaware of their status and it is further estimated that over half of all new infections are acquired from someone who is unaware of their infection [2], [3]. Identification of HIV is the requisite first step toward treating HIV and reducing HIV-1 VL levels. Since lower VL levels are associated with decreased risk for sexual transmission [4], [5], timely ART may be an effective way of reducing new infections [6], especially if a large proportion of people living with HIV/AIDS (PLWHA) access treatment early in disease progression [2]. Most screening in North America, however, does not diagnose HIV until the disease is advanced. In 2007, the median value for the first CD4 count after diagnosis in the United States was 177 cells/mL [7]. A model assessing the impact of increased treatment coverage on transmission dynamics in at-risk populations in British Columbia predicts a dramatic reduction in new cases between 2006 and 2030 if ART coverage is increased above its current level of 50% among individuals with CD4 counts <200 cells/mL [8]. Granich et al. used South African data to model an idealized intervention, where frequent testing and immediate treatment of 90% of infections would lead to substantial reductions in new infections [9]. Although other authors using “more realistic” models question Granich et al. that expanded ART could eliminate the HIV epidemic, they agree that more widespread ART will effectively reduce HIV transmission [10].

Examining how the criminal justice system (CJS) might be leveraged not only to detect new HIV infections but also engage HIV-infected persons in care has not yet been fully explored; however, there is clear evidence that jails can be effective sites to diagnose new infections and to begin to implement routine care and prepare for transitional healthcare delivery [11]. The CJS is a particularly important site to address the HIV epidemic in the U.S., since drug and alcohol abuse contribute to ongoing HIV transmission and are highly prevalent in CJS populations [12], [13], [14].

Each year, one in six PLWHA in the U.S. spends time within a CJS facility. Over 90% of persons entering and leaving U.S. correctional facilities spend time in jails, which are short-term facilities for persons awaiting trial or sentenced for brief periods of time, and do not move on to prison [15]. Since most jail inmates return to the community rather than move on to a prison, jails can help with continuity of care by having effective transitional programs for HIV-infected detainees [16].

Former inmates who are HIV-infected, including those who either are not initiated on ART or who do not remain on it, often engage in ongoing HIV risk behaviors, which can contribute to community HIV transmission [17]. Recent data from the HIV Prevention Trials Network (HPTN 052) support the use of ART to suppress viral replication and reduce heterosexual HIV transmission to HIV seronegative sexual partners [18]. ART use among releasees is critical to reduce transmission to sexual and drug-using community partners. Recent studies have shown the transition period from incarceration to community resettlement to be a time of particularly high risk for cessation of ART with multiple barriers to ART adherence and filling ART prescriptions [19], which results in rebounded viral loads [20] and may, in turn, result in increased risk of transmission to sexual partners.

In 2007 the U.S. Health Resources and Services Administration's (HRSA) HIV/AIDS Bureau awarded 10 grants through their Special Projects of National Significance (SPNS) program to organizations through the Enhancing Linkages to HIV Primary Care and Services in Jail Settings Initiative (EnhanceLink). This initiative sought to implement and evaluate models for linkages to healthcare for PLWHA who were leaving jails. HIV testing has been an important aspect of the project; over 180,000 HIV tests have been conducted across the 10 sites.

As part of the multisite evaluation of the project [21], we conducted a substudy to assess baseline HIV characteristics and use of ART among individuals who were newly diagnosed with HIV in jail and compared them to community-derived samples. We also assessed linkage to community HIV care among those released from jail. The overall aim was to provide a detailed description of a subset of new diagnoses within this setting. A secondary aim was to statistically compare them demographically and clinically to those who had previously been diagnosed within this cohort.

## Methods

### Ethics statement

The Institutional Review Boards of Rollins School of Public Health of Emory University and Abt Associates approved the multisite study. Subsequently, the 10 individual sites' Institutional Review Boards approved their individual study involvement. A certificate of confidentiality was also obtained for the study.

### Methodology and analysis

The EnhanceLink study provides an opportunity to gauge how effective the jail setting can be for the early diagnosis of HIV followed by prompt linkage to medical care. The 10 diverse EnhanceLink jail demonstration programs have been previously described [21]. Briefly, demonstration sites are located in: Atlanta, GA; Chester, PA; Chicago, IL; Cleveland, OH; Columbia, SC; New Haven, CT; New York, NY; Philadelphia, PA; Providence, RI; and Springfield, MA. Most of the jails in EnhanceLink offer some routine, if not universal, HIV testing.

The EnhanceLink project is collecting two types of information on project activity. First, each site collected program-level data on a quarterly basis from the inception of their involvement in the project through March 31, 2011. Second, the portion of the project highlighted here, was the collection of client-level data (CLD) from approximately 1 in 6 participants. These individuals were approached and asked to participate in a voluntary evaluation of their experience in the linkage programs. Sites varied in criteria for enrollment in the client-level evaluation. While all sites limited enrollment to persons 18 years or older, one site (New York, NY) only enrolled persons in the CLD portion of the evaluation whose diagnosis was made prior to their most recent jail admission. CLD from the 9 sites eligible for this analysis of newly diagnosed individuals are available on 781 individuals. Evaluators administered a baseline survey to consenting detainees that included questions on demographic characteristics such as age, sex and race. Five of the nine sites asked supplemental questions on sexual risk behavior at the baseline interview. Clients were initially classified as newly diagnosed if they affirmed on the baseline survey that their HIV diagnosis was made during their current jail stay. After the client was released from their index jail stay, clinical data including CD4 count and plasma VL as measured in commercial laboratories and conducted in the course of clinical care were extracted from jail medical records. Six months after release from their index jail stay, the clients participated in a follow-up survey. For the present analysis of newly diagnosed clients, we confirmed that there was no evidence of a prior HIV diagnosis from the jail medical record; we reclassified clients as previously diagnosed if the chart referred to HIV-related laboratory tests drawn in the community prior to index incarceration. The first CD4 count after diagnosis was examined as well as whether ART was started before jail discharge and whether a genotype was requested and reviewed before initiating ART. Linkage to community HIV care, defined as having either a VL, CD4 count or both measured within the 6 months post release from the index incarceration was ascertained from case management records and review of medical records from community clinics. The present analysis used data submitted by sites by August 31, 2011; clients who were deemed ineligible after this date were removed from the analysis.

The first objective was to describe the CLD of the newly diagnosed population; descriptive statistics were used. The second objective was to compare client-level data from the newly diagnosed detainees to client-level data obtained from detainees diagnosed prior to their current jail incarceration. Comparisons with those previously diagnosed were carried out using a two-sample t-test for continuous outcomes and Pearson chi-square test for discrete outcomes. Exact tests (Fisher's) were used where we had observations on fewer than 5 clients. All analyses were done in SAS version 9.2 (SAS Institute, Cary, North Carolina).

## Results

Across the ten demonstration sites, 210,267 jail detainees were screened for HIV and 1,312 (0.62%) of tests returned positive; 822 of the diagnoses were new. The proportion of newly diagnosed clients among detainees screened was not uniform across the sites. Some sites were significantly more likely (p<0.01) to have tests yielding new diagnoses. The proportion of positive tests at each site that were new diagnoses ranged between 0.12% and 1.38%. Program-wide, about 1 in 6 of all persons in the jails who were known to be HIV positive, including 58 new diagnoses as well as those already aware of their HIV infections, agreed to participate in more extensive, individual level evaluation. To our knowledge, this is the largest program following a group of patients diagnosed in jail with concurrent individual level data collection.

### Demographics

Of the 781 HIV-positive detainees for whom CLD were collected, we have information on date of diagnosis for 766. Of these 766, 43% (N = 329) were diagnosed initially within a correctional facility, including the 58 detainees who were first diagnosed during their current jail stay (See Table 1). The median age of those newly diagnosed was 33 years. The newly diagnosed jail detainees were 62% male (N = 36), 67% Black (N = 39) and 12% (N = 7) Hispanic ethnicity. The clients who agreed to provide CLD had a median jail length of stay of 75 (IQR: 31–147) days. Data on individual risk behavior were available for 60% (N = 35) newly diagnosed participants and 53% (N = 372) previously diagnosed participants; demographics did not differ significantly among newly and previously diagnosed participants who provided risk behavior data. Among newly diagnosed persons, 4 of 35 (11%) reported a history of injection drug use in the 30 days prior to index incarceration. Among newly diagnosed men, 7 of 37 (19%) reported a homosexual or bisexual orientation. Of 24 men completing the risk behavior component of the survey, 6 (25%) reported having sex with men in the last 30 days.

**Table 1:** Internal comparison of newly diagnosed and previously diagnosed EnhanceLink jail detainees.

	Newly Diagnosed	Previously Diagnosed	p-value
	N (%)	N (%)	
**Total**	58 (8)	708 (92)	
**Mean age (yrs)**	34 (SD = 10)	41 (SD = 9)	<0.01
**Median age (yrs)**	33	42	
**Black Race**	39 (67)	436 (64)	0.59
**Hispanic ethnicity**	7(12)	120 (18)	0.31
**Male gender**	36 (62)	466 (67)	0.59
**Median length of stay in jail (days)**	68	76	
**Among males:**			
**Self-identified as bisexual or homosexual**	7 (19)	102 (22)	0.71
**Reports sex with men in the past 30 days** +	6 (25)	26 (12)	0.08
**Among both males & females:**			
**Reported unsafe anal or vaginal sex in the past 30 days** +	21 (60)	96 (27)	<0.01
**Reported unsafe anal sex in the past 30 days** +	10 (29)	34 (9)	<0.01
**Reported unsafe vaginal sex in the past 30 days** +	15 (44)	87 (24)	0.01
**Reported sex with someone other than main sex partner in past 30 days** +	18 (51)	89 (24)	<0.01
**Reported use of needles to inject drugs in past 30 days**	4 (11)	42 (12)	0.99
**In a committed relationship**	18 (31)	225 (32)	0.88
**Most common employment situation in the past 3 yrs was any kind of work**	24 (42)	180 (26)	<0.01
**Homeless at index incarceration**	25 (43)	311 (44)	0.88
**Had some health insurance** ++ **or benefits to pay for all or part of medical care**	20 (35)	462 (66)	<0.01
**Started or restarted ART while in jail**	12 (24)	212 (38)	0.04
**Reported any emergency room visits in the last 6 months**	23 (40)	346 (50)	0.15
**Any indication of linkage to community care**	26 (74)	335 (68.)	0.46

+ Obtained from risk behavior module completed by 59% (N = 35) of those newly diagnosed and 52% (N = 372) of those previously diagnosed.

++ Insurance refers to all health insurance, not just HIV-related health insurance.

### Clinical Status

The median CD4 count of the 58 newly diagnosed subjects was 432 (range 22–1,453) cells/mL; 64% had a CD4 count below 500 cells/mL, within the range where DHHS guidelines have become increasingly more supportive of initiation of ART [22]. Fourteen percent had an initial CD4 count ≤200 cells/mL, requiring prophylaxis for opportunistic infections; 24% had an initial CD4 count of 201–350 cells/mL and 26% had a CD4 count of 351–500 cells/mL. Of the 58 new diagnoses, 21% (N = 12) were started on ART while in jail, 67% (N = 39) were not and 12% (N = 7) were missing ART data. The median length of stay for the newly diagnosed who started ART while in jail was 160 days (IQR: 111–204 days) three times the median length of stay (49 days) for the newly diagnosed who did not start ART while in jail. Of the 12 clients started on ART, an HIV genotype was present in the chart of only 3 (25%) individuals. All 3 of these persons had a length of stay >2 months. Those who were initiated on ART were significantly more likely (p<0.01) to have both a CD4 count below 350 cells/mL and a length of incarceration exceeding 2 weeks compared to those who did not start ART. Of the 35 newly diagnosed who had been released for at least 6 months and for whom a six-month follow-up would be expected, 74% (N = 26) had some evidence of medical visits in the community.

### Comparison of newly diagnosed and previously diagnosed

As shown in Table 1, the mean age of those newly diagnosed (34 years) was significantly younger (p<0.01) than those previously diagnosed (41 years). The newly and previously diagnosed did not differ in terms of gender, race or ethnicity. There were, however, significant differences between newly and previously diagnosed when comparing recent sexual risk taking behavior. Newly diagnosed clients were more likely to have reported sex with someone other than their main partner (p<0.01), unsafe vaginal sex (p = 0.01), and unsafe anal sex (p<0.01) in the 30 days prior to index incarceration as compared to their previously diagnosed peers.

Newly diagnosed persons were more likely (p<0.01) to have a history of being employed in the 3 years prior to index incarceration. A similar proportion of those newly diagnosed (40%) and of those previously diagnosed (50%) reported visiting an emergency room within the last 6 months. Newly diagnosed clients were significantly less likely (p<0.01) to have health insurance at baseline (health insurance defined as all forms of health benefits, not just HIV-related benefits) when compared to those previously diagnosed. There was no significant difference in the likelihood of having completed a 6-month follow-up medical appointment, or being linked to medical care post release for those newly diagnosed (74%) compared to the previously diagnosed detainees (68%, see Table 1).

Among those previously diagnosed, 78% (N = 538) had ever taken HIV medications and 55% (N = 306) were on HIV medication 7 days prior to their incarceration. About one-third (38%, N = 212) of those previously diagnosed started or restarted ART while in jail, which was significantly higher (p = 0.04) higher than the proportion starting ART among those newly diagnosed (24%, N = 12) (See Table 1).

## Discussion

Results from the EnhanceLink demonstration project suggest that HIV testing in jails can lead to new diagnoses of HIV infection and that these infections are being diagnosed substantially early on in the course of the disease. Among the 781 HIV positive inmates with CLD, the newly diagnosed represent 8% (N = 58). Irrespective of time of HIV diagnosis, nearly one half (44.3%) of the clients was first diagnosed with HIV in a correctional facility, either a jail or a prison. The “correctional origin” of the HIV diagnosis is consistent with other studies demonstrating the important public health benefit of routine HIV testing in jails [23], [24]. Previous reports have indicated that only a minority of jail facilities offer routine HIV testing [25], [26]. Our findings suggest that situating testing in jails is feasible and is associated with HIV detection at an early stage. Implementing voluntary, opt-out HIV testing in more jails in those regions of the country most profoundly impacted by the HIV epidemic is consistent with public health needs.

Based on data from this study, the rapid turnover of jail detainees is not an impediment to jail-based HIV screening programs. HIV testing can still be accomplished for large numbers of detainees. Across all of the EnhanceLink demonstration projects, including New York, NY, over 180,000 detainees were screened in the 30 month period between September 2008 and March 2011. Correctional facilities that have implemented opt-out testing show substantially increased percentages of individuals tested [24]. Five of 10 sites in the EnhanceLink project have had at least one participating jail offer routine opt-out HIV testing at some point during the project period.

What would be the result of improving availability of HIV testing in jail facilities across the U.S.? CDC strongly recommended the implementation of jail-based testing in their 2009 guidance on HIV testing in correctional settings [27]. At the EnhanceLink demonstration sites that provide laboratory data on newly diagnosed persons, the initial median CD4 count of 432 cells/mL was markedly higher than the median count of the first CD4 test in 37 states reporting state-wide HIV data to the CDC (177 cells/mL) [7]. Additionally, the median CD4 cell count of newly diagnosed detainees in EnhanceLink is greater than the median CD4 count at presentation estimated from the North American-AIDS Cohort Collaboration on Research and Design (NA-ACCORD) in 2007 (317 cells/mL) [28].

The median first CD4 count after diagnosis varies by testing venue. Table 2 shows median first CD4 counts published in the literature. The reported median CD4 obtained from individuals screened in the emergency department context [29], [30] was lower than detainees diagnosed in the EnhanceLink jails. In contrast, routine testing in a VA Medical System yielded a mean CD4 count of 393 cells/mL [31]. Routine HIV testing in the Washington State Prison system found that initial CD4 counts averaged 422 cells/mL [24]. While testing in prisons, like jails, finds persons early in disease, confining testing just to prisons may have less impact than testing in all adult correctional facilities, since seventeen-fold more individuals pass through jails than prisons [15].

**Table 2:** Published median CD4 counts and median age at first HIV diagnosis.

Median CD4 count	Median Age	(Year) Study population & design
(cells/mL)	(yrs)	
356	-	(2005–2006) As part of a CDC-funded demonstration project, HIV screening was offered to medically stable patients aged 12 years or older in an urban emergency department located at the Alameda County Medical Center [30].
324	33	(1998–2003) Visit records were reviewed for a cohort of patients who received a new HIV diagnosis between July 1999 and June 2003. Patients were recruited from an urban academic, an urban community and a suburban community emergency department located within 10 miles of one another [29].
317	41	(1997–2007) Data were analyzed from 44,491 HIV-infected patients enrolled in the North American-AIDS Cohort Collaboration on Research and Design identified at first presentation for HIV care. The NA-ACCORD is a multisite collaboration of 8 interval and 14 clinical cohort studies in the U.S. and Canada [28].
220	36	(2006–2008) Study population consisted of individuals 18 years or older and newly diagnosed with HIV, who had a genotype done between January 2006 and December 2008 and entered care for the first time at the Henry Ford Hospital located in downtown Detroit [46]
177	-	(2007) Data compiled from 37 states with HIV reporting to the Centers for Disease Control and Prevention (CDC) for at least 4 years. The median CD4 count was compiled from the first CD4 test performed within 3 months after diagnosis of HIV infection [7].
53	38	(1998–1999) Study population consisted of consecutive newly diagnosed HIV-infected patients from all inpatient and outpatient HIV tests performed at the Harbor-UCLA Medical Center in California. This hospital serves a primarily urban minority population including patients at high risk of HIV infection [47].

Forty percent of those newly diagnosed persons in our cohort reported visiting an emergency room at least once in the 6 months prior to baseline, suggesting that screening within jails can lead to detection of undiagnosed HIV that may have been missed in other settings. Consistent with earlier detection, the demonstration sites are also finding HIV-infected persons at a younger age than found in other settings. The median age at first diagnosis within the demonstration sites is substantially younger than the median age at first diagnosis for the general North American population enrolled in a consortium of research studies [28].

Identifying HIV infection at an earlier stage is of high priority as it has significant implications for reducing potential transmission to drug using and sexual partners and to improving individual health. Routine HIV screening in jails remains consistent with the National HIV/AIDS Strategy of increasing the proportion of PLWHA who know their serostatus from 79% to 90% [32]. The earlier individuals are aware of their status, the sooner they can be linked to care and services that would reduce onward transmission within their communities. Identification of HIV alone has been associated with a 3–4 fold reduction in sexual risk behaviors even before implementing targeted interventions [33], [34]. Regarding improved individual health, a recently developed computer simulation model showed that for persons infected in 2010, diagnosing HIV early, when the median CD4 count was 432 cells/mL, rather than late, when median CD4 count had dropped to 140 cells/mL, was associated with 3.5 years greater life expectancy [35]. Others have shown that when patients present early, hospitalizations are less frequent and lower costs per patient persist for over seven years [36], [37].

The newly diagnosed individuals in our cohort represent a riskier group with regards to sexual risk taking behavior than those already aware of their diagnosis. Newly diagnosed clients were significantly more likely to have reported sex with someone other than their main partner, unsafe vaginal sex, and unsafe anal sex in the 30 days prior to index incarceration as compared to their previously diagnosed peers. This is consistent with evidence indicating reductions in risky behavior among individuals who have been diagnosed and are aware that they are HIV-infected [33], [34]. The heightened risky behavior further emphasizes the need to identify these individuals as early as possible in order to reduce transmission to others by initiating counseling and ART.

While diagnosing individuals within the CJS represents an effective public health approach to early detection and HIV prevention, it likely raises questions within correctional facilities regarding costs of treating HIV-infected patients once they are identified. Within this project, most newly diagnosed individuals did not initiate ART before discharge. There is no evidence that identifying HIV-infected persons substantially increased pharmacy costs in this project: approximately 50% of jail detainees are released in two days [38] and thus most newly diagnosed persons may leave the facility before a comprehensive pre-treatment evaluation can be completed. The ideal situation is not to detain a person in jail longer than legally necessary to initiate ART; the goal of the EnhanceLink projects was to assure linkage to medical care, including treatment when indicated, following jail.

For the small percentage of patients who remain in jail long enough to complete testing and evaluation for the appropriateness of ART, genotyping, initiation of ART and the subsequent steps of care provision may also be feasible to begin in jail. Genotype testing is recommended by the DHHS guidelines before initiating therapy to identify baseline ART resistance, which affects the initial choice of ART medications [22]. The majority of newly diagnosed persons in our cohort who initiated ART before jail release, however, did not have genotype testing prior to starting treatment. Taking the time to obtain a genotype may decrease the number of persons who initiate care before discharge. With such a large proportion of positive detainees having very short lengths of stay, the increased cost to jails of offering ART to longer term detainees should be relatively minimal while making an important contribution to reducing community viral load. The critical step for linkage to care, which is the identification of previously undiagnosed HIV, can begin within jails.

Data on HIV medication either over one's lifetime or in the 7 days preceding incarceration suggest that the majority of those previously diagnosed have come off ART prior to incarceration [39]. Jails therefore serve as an opportunity to re-start ART among those who have fallen out of care or have come off ART due to substance use. Data from San Francisco jails show that, among HIV-positive persons involved in the criminal justice system, those who use ART only in jail have higher CD4 counts and lower viral loads compared to those who never use ART [40].

In terms of challenges regarding linkage to care faced by case managers, not only do they have to support the newly diagnosed who are coming to terms with their HIV status, but they are also charged with re-linking those previously diagnosed to care. Our result showing that 74% of the newly diagnosed had linkage to HIV care in the community and that the previously diagnosed had a similar linkage rate demonstrates that EnhanceLink case managers, in innovative partnership with jails, did an admirable job linking both population groups to care, despite the challenges [21].

There are limitations to this study. Data regarding risk behaviors is cross-sectional, limiting any causal inference—it is difficult to ascertain temporality. Apart from the laboratory results, the data are largely verbally self-reported to case-workers or medical professionals and therefore are subject to social desirability bias. The population of detainees who voluntarily provided CLD is a biased subset skewed towards those with lengths of stay, who may have more in common with long-term prisoners than the typical short-term jail detainees; this subset of detainees may not be representative of HIV-positive jail detainees in general. Yet the suitability of using the prison setting for HIV interventions is already well-established [41], [42]. Last, while we did report a relatively high linkage to community services after release based on our data, further research is needed to determine to what extent these newly diagnosed persons accessed ART and remained retained in HIV treatment.

Despite these limitations this is the largest program, to our knowledge, with concurrent individual level data on jail detainees compiled from 9 sites located in different geographic regions across the U.S. with characteristically different HIV epidemics. The wide range in the proportion of new diagnoses of all tested among sites most likely reflects differences in HIV prevalence, stage of the epidemic, testing policies within correctional facilities as well as previous availability of testing services within the region, since yield of testing programs can vary with time [43].

In conclusion, jails remain key sites for diagnosing individuals at early stages of infection. Public health partnerships, linked to funding resources, may help to mitigate some of the challenges to implementation. For example, many of the new criminal justice “seek, test, and treat” models being implemented and tested around the country are multisectoral partnerships that include academic medical centers, community-based organizations, and correctional facilities and clinics [44], [45]. Each type of organization brings to the table needed skills and expertise in identifying, testing, treating and retaining HIV-infected individuals in care.

The linkage process for jail detainees who are newly diagnosed HIV positive will be enhanced by evidence-based interventions. While the multiple needs of those passing through the CJS are common to individuals in other settings, they may be more pronounced among those who have been incarcerated. Domains of need include, in addition to HIV primary care, housing, transportation, mental health and substance abuse treatment. Developing effective programs and seamless services that address these needs will have the best chance of keeping individuals in care. In summary, testing in jails is feasible, pharmacy costs for jails may be manageable and linkage to care upon discharge is achievable.
